# Viral Surveillance in Serum Samples From Patients With Acute Liver Failure By Metagenomic Next-Generation Sequencing

**DOI:** 10.1093/cid/cix596

**Published:** 2017-07-19

**Authors:** Sneha Somasekar, Deanna Lee, Jody Rule, Samia N Naccache, Mars Stone, Michael P Busch, Corron Sanders, William M Lee, Charles Y Chiu

**Affiliations:** 1 Department of Laboratory Medicine, University of California San Francisco (UCSF) and; 2 Blood Systems Research Institute, San Francisco, California;; 3 Department of Medicine, Division of Digestive and Liver Diseases, University of Texas Southwestern Medical Center, Dallas; and; 4 UCSF–Abbott Viral Diagnostics and Discovery Center and; 5 Department of Medicine, Division of Infectious Diseases, University of California San Francisco

**Keywords:** pathogen discovery, metagenomic next-generation sequencing, indeterminate ALF, viral hepatitis, SURPI computational pipeline

## Abstract

**Background:**

Twelve percent of all acute liver failure (ALF) cases are of unknown origin, often termed indeterminate. A previously unrecognized hepatotropic virus has been suspected as a potential etiologic agent.

**Methods:**

We compared the performance of metagenomic next-generation sequencing (mNGS) with confirmatory nucleic acid testing (NAT) to routine clinical diagnostic testing in detection of known or novel viruses associated with ALF. Serum samples from 204 adult ALF patients collected from 1998 to 2010 as part of a nationwide registry were analyzed. One hundred eighty-seven patients (92%) were classified as indeterminate, while the remaining 17 patients (8%) served as controls, with infections by either hepatitis A virus or hepatitis B virus (HBV), or a noninfectious cause for their ALF.

**Results:**

Eight cases of infection from previously unrecognized viral pathogens were detected by mNGS (4 cases of herpes simplex virus type 1, including 1 case of coinfection with HBV, and 1 case each of HBV, parvovirus B19, cytomegalovirus, and human herpesvirus 7). Several missed dual or triple infections were also identified, and assembled viral genomes provided additional information on genotyping and drug resistance mutations. Importantly, no sequences corresponding to novel viruses were detected.

**Conclusions:**

These results suggest that ALF patients should be screened for the presence of uncommon viruses and coinfections, and that most cases of indeterminate ALF in the United States do not appear to be caused by novel viral pathogens. In the future, mNGS testing may be useful for comprehensive diagnosis of viruses associated with ALF, or to exclude infectious etiologies.

## Teaser

Viral metagenomic surveillance of 187 patients with indeterminate acute liver failure (ALF) from 1998 to 2010 suggests that these patients should be screened for the presence of uncommon viruses and coinfections, although novel viruses associated with ALF were not identified.

**(See the Editorial Commentary by Fredricks on pages 1486–8.)**

Acute liver failure (ALF) is a complex syndrome characterized by rapid deterioration of liver function with hepatic encephalopathy in the absence of a known history of previous liver disease. ALF may progress to multiorgan failure and results in death or transplantation in 57% of cases. Acetaminophen toxicity is the most common documented cause of ALF in the United States, comprising 50% of cases, while infections from hepatitis A virus (HAV) and hepatitis B virus (HBV) combined account for 12% [1]. After standard clinical testing, a clear etiology is not found in approximately 12% of US ALF cases, thus considered indeterminate; an even higher percentage of indeterminate cases (~20%) is seen in developing countries [2]. Infection by a previously unrecognized hepatotropic virus has been posited as a cause for indeterminate ALF [3]. Beyond HAV and HBV, other potential viral etiologies of ALF include hepatitis C virus (HCV), hepatitis E virus (HEV), adenovirus, herpes simplex virus type 1 (HSV-1), Epstein-Barr virus (EBV), varicella zoster virus, and parvovirus B19, all of which are rare causes of ALF in the United States [4–7]. Appropriate management of ALF depends on accurate diagnosis, as specific antiviral treatments are sometimes available, and other therapies may be indicated for noninfectious etiologies [7]. Thus, it is imperative to determine the limitations of conventional diagnostic testing for ALF, and whether novel or previously unrecognized viruses may be associated with this condition.

Metagenomic next-generation sequencing (mNGS) is a comprehensive approach for sequence-based identification of pathogenic microbes, especially viruses, in clinical samples [8, 9]. Previous studies have shown that mNGS is useful for viral surveillance [10, 11] and identification of novel viruses circulating in blood [12, 13]. The US Acute Liver Failure Study Group (ALFSG) is a nationwide study established in 1998 to enroll patients with ALF and collect detailed clinical and laboratory information and biosamples for analysis. We hypothesized that mNGS screening of serum samples from 187 indeterminate ALFSG cases from 1998 to 2010 would allow broader identification of infectious causes of indeterminate ALF.

## METHODS

### Study Patients

A total of 204 patients with ALF (187 patients with indeterminate ALF and 17 as controls) were selected from the ALFSG cohort (Supplementary Methods) to undergo further mNGS testing for viral infection. Clinical and laboratory data for the 204 ALF patients, including available liver biopsy data and history of injection drug and alcohol use, are provided in Supplementary Tables 1–4. The 187 indeterminate ALF cases were consecutive (collected during enrollment years 1998–2010), provided a broad geographic representation across all study sites in the United States, and comprised 61% of the overall cohort to date (n = 307). Notably, 11 of the indeterminate samples tested positive for viruses at the study site, including human immunodeficiency virus (HIV) (n = 2), chronic HCV (n = 3), mixed HBV, HCV, and hepatitis D virus (HDV) (n = 1), combined EBV and cytomegalovirus (CMV) (n = 1), and HBV (n = 4). The 17 control patient samples included 8 samples from ALF-associated hepatitis A or B cases (n = 4 each) and 9 samples corresponding to noninfectious negative control groups: acetaminophen (*N*-acetyl-para-aminophenol [APAP]) toxicity (n = 3), autoimmune hepatitis (n = 2), drug-induced liver injury (n = 3), and hepatic ischemia (n = 1).

### Metagenomic Next-Generation Sequencing and Analysis

Serum samples from each patient were processed in a blinded fashion for metagenomic sequencing individually or in pools of 2–6 (Supplementary Table 5), using an approach demonstrating high sensitivity for unbiased virus detection [14]. Individual samples or pools were first treated with a cocktail of Turbo DNase (Ambion) and Baseline-ZERO DNase (Epicentre) prior to nucleic acid extraction. Pretreatment with DNase biases mNGS testing toward viral detection as microbial and human host DNA is depleted whereas encapsidated (“protected”) viral nucleic acid is preserved [15]. With analysis of serum, there is also a minor limitation with respect to potential decreased sensitivity of detection of integrated proviruses (eg, HIV type 1 [HIV-1]), episomal viruses (eg, herpesviruses), or strongly cell-associated viruses (eg, human T-cell lymphotropic virus 1) [16]. Nucleic acid extraction was performed on the Qiagen EZ1 Advanced XL automated system using the EZ1 Virus Mini Kit version 2.0 (Qiagen). Extracted nucleic acid was amplified with random hexamers to generate a complementary DNA (cDNA) library as previously described [17]. In brief, 23 pools and 12 individual samples were initially processed using a modified TruSeq protocol (Illumina) [18], and 116 individually prepared samples were later processed using the NexteraXT protocol (Illumina). Samples were sequenced on 10 Illumina HiSeq lanes, and sequencing reads were analyzed using sequence-based ultra-rapid pathogen identification (SURPI), a computational pipeline for comprehensive pathogen identification from mNGS data by comparison to microbial sequences in the National Center for Biotechnology Information (NCBI) nucleotide (NT) database [9]. SURPI+, the clinical version of SURPI used for analysis [18, 19], employs taxonomic classification according to a lowest common ancestor algorithm for more accurate read assignments. As the first set of TruSeq libraries used only single-end barcode indexes (vs paired-end barcodes for the NexteraXT libraries), a predetermined threshold of >25 reads and >2% genome coverage was used for assessment of positive viral signatures by mNGS in these single-end barcoded samples given the potential for cross-contamination. Detected reads from viruses of unknown pathogenicity that constitute part of the normal viral flora circulating in blood, such as anelloviruses and human pegivirus 1/GB virus C [20, 21], were not considered as causes of ALF (Supplementary Table 6). Manual analyses for bona fide reads corresponding to nonviral pathogens (bacteria, fungi, and parasites) revealed only the presence of known laboratory/environmental contaminants or false-positive identifications due to misannotations in the NCBI NT database.

Details regarding genome assembly, genotyping, and identification of resistance mutations, as well as sequencing data deposition into the NCBI Sequence Read Archive, are provided in the Supplementary Methods.

### Confirmation by Research Nucleic Acid Testing

Individual samples or all samples comprising a pool were selected for research nucleic acid testing (NAT) confirmation if reads from a human viral pathogen (HAV, HBV, HCV, HDV, HEV, CMV, HIV, parvovirus B19, and human papillomavirus virus type 159 [HPV-159]) were detected by mNGS. Total nucleic acid was extracted using the Qiagen Ultrasens Virus Kit (Qiagen), followed by construction of cDNA libraries using random hexamers and Superscript III Reverse Transcriptase (Invitrogen). Libraries were screened by polymerase chain reaction (PCR) (Qiagen OneStep RT-PCR Kit) using previously published primer sets and conditions for detection of individual viruses (Supplementary Table 7).

### Confirmation by Clinical Nucleic Acid Testing

In parallel, NAT was independently performed on 0.5 mL serum from samples testing positive by mNGS and with sufficient volume available using the Procleix Ultrio assay on the Tigris platform (Grifols Diagnostic Solutions and Hologic). This US Food and Drug Administration–approved clinical assay is employed in the United States and internationally for screening blood donors for simultaneous detection of HIV types 1/2 and HBV and HCV nucleic acids [22, 23]. The analytic sensitivities of Ultrio for detection of HIV, HCV, and HBV are 10–15 copies or IU/mL at a 95% lower limit of detection.

## RESULTS

### Clinical Testing

**Figure 1.**
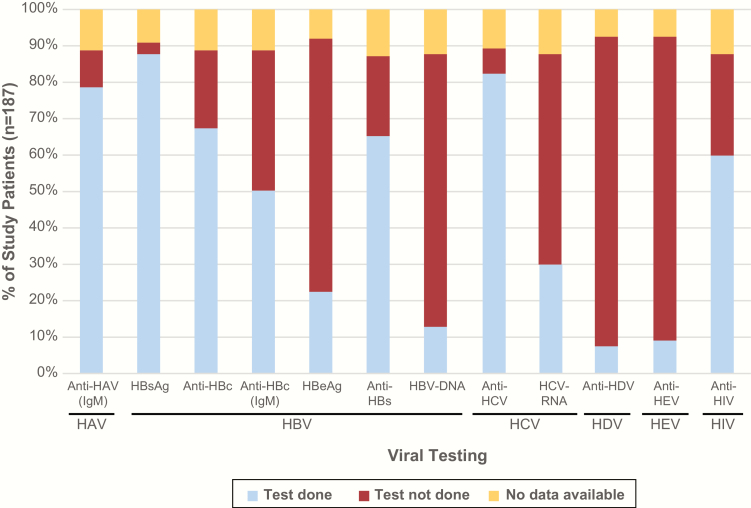
Clinical diagnostic testing for viral infections in study patients with indeterminate acute liver failure. The percentage of patients in the study positive for a given viral infection (y-axis) is plotted against the specific laboratory test that was ordered by the clinical site (x-axis). Abbreviations: anti-HBc, hepatitis B core antibody; anti-HBs, hepatitis B surface antibody; HAV, hepatitis A virus; HBeAg, hepatitis B e antigen; HBsAg, hepatitis B surface antigen; HBV, hepatitis B virus; HCV, hepatitis C virus; HDV, hepatitis D virus; HEV, hepatitis E virus; HIV, human immunodeficiency virus; IgM, immunoglobulin M.

Serum samples from 204 patients were investigated in a blinded fashion using mNGS, of which 187 (92%) were classified as indeterminate, 8 (4%) were positive controls, and 9 (4%) were negative controls (Table 1). The extent of primary clinical testing for hepatitis viruses at the study sites was variable, with HCV serology and HBV antigen testing being performed >80% of the time, and HDV or HEV PCR testing <10% of the time (Figure 1).

**Table 1.** Cause of Acute Liver Failure at Time of Hospital Discharge for Patients in the Study (N = 204)

Cause of Acute Liver Failure^a^	Samples, No. (%)
Indeterminate	187 (91.7)
Hepatitis A virus infection^b^	4 (2.0)
Hepatitis B virus infection^b^	4 (2.0)
Acetaminophen toxicity^c^	3 (1.5)
Autoimmune hepatitis^c^	2 (1.0)
Drug-induced hepatitis^c^	3 (1.5)
Hepatic ischemia^c^	1 (0.5)

^**a**^An “indeterminate” diagnosis is given to patients when the cause of acute liver failure is unclear at time of hospital discharge.

^b^Viral positive control.

^c^Noninfectious negative control.

### Metagenomic Next-Generation Sequencing Results

Serum samples collected at the earliest available time point after clinical presentation of ALF were analyzed (median, 7 days [range, 2–38 days] from symptom onset to sample collection; Table 2). A total of 151 individual or pooled sample libraries from 204 serum samples were sequenced across 10 Illumina HiSeq lanes, yielding 1.5 billion raw sequence reads. After preprocessing to remove low-complexity and low-quality sequences, 84% of the remaining preprocessed reads were classified as human and 1.0% as viral by SURPI+ (Supplementary Table 8). The 12 million viral reads included canonical hepatitis viruses (HAV, HBV, HCV, and HDV) and additional viruses rarely associated with hepatitis, such as HSV-1 and parvovirus B19 (Table 2). Reads from anelloviruses and/or human pegivirus 1, considered nonpathogenic flora [20, 21], were identified in 135 of 151 (89.4%) sequencing libraries.

**Table 2.** Acute Liver Failure Patients With Serum Positive for a Virus By Metagenomic Next-Generation Sequencing and/or Confirmatory Nucleic Acid Testing

Patient ID	Initial Site Diagnosis	Clinical Site Testing												Illness Onset to Sample Collection for mNGS, d	mNGS Result	UCSF Lab Research NAT	Ultrio NAT	ALF Etiology^b^
		HAV IgM	HBsAg	Anti-HBc	Anti-HBc IgM	HBeAg	Anti- HBs	HBV DNA	Anti- HDV	Anti- HCV	HCV RNA	Anti- HEV	Anti- HIV					
10–2006^c^	HAV	+	–	ND	–	ND	ND	ND	ND	–	ND	ND	ND	9	HAV +	HAV +	ND	HAV
13–2207^c^	HAV	–	–	ND	–	ND	–	ND	ND	+	+	ND	–	11	HAV +	HAV +	ND	HAV
15–2455^a,^^c^	HAV	+	–	–	–	–	–	–	–	–	ND	ND	–	7	HAV +	HAV +	ND	HAV
23–2911^c^	HAV	+	–	–	ND	ND	–	ND	ND	–	ND	ND	–	9	HAV +	HAV +	ND	HAV
14–2440^c,d^	Hepatitis B	–	+	+	+	+	–	+	ND	–	ND	ND	ND	5	HBV +	HBV +	ND	HBV
18–2652^c^	Hepatitis B	–	+	+	+	+	–	+	–	–	–	ND	–	5	HBV –	HBV +	ND	HBV
18–2653^a,^^c^	Hepatitis B	–	+	+	–	+	+	+	–	–	ND	+	ND	21	HBV +	HBV +	ND	HBV
33–3251^c^	Hepatitis B	–	+	+	+	+	+	+	ND	–	ND	ND	–	2	HBV +, HSV-1 +	HBV +, HSV-1 +	ND	HBV and HSV-1 coinfection
10–2053^e,f^	Indeterminate^g^	–	+	–	–	ND	–	ND	–	–	–	ND	–	4	HBV +	HBV +	+	HBV
13–2383^e,h^	Indeterminate^g^	ND	+	+	–	ND	+	+	–	–	ND	ND	–	7	HBV +	HBV +	+	HBV
21–2833^e,i^	Indeterminate^j^	–	–	ND	ND	ND	+	ND	ND	–	ND	ND	ND	6	HBV –	HBV +	ND	HBV
35–3354^e^	Indeterminate^k^	–	+	–	+	ND	–	ND	ND	–	–	ND	ND	5	HBV +	HBV +	+	HBV
11–2071^e,l^	Indeterminate	–	+	–	–	–	–	ND	+	+	+	ND	ND	7	HBV –, HCV +, HDV +	HBV +, HCV +, HDV +	+	HBV, HCV, HDV coinfection
10–3875^e,m^	Indeterminate	–	–	ND	–	ND	–	ND	ND	+	+	–	–	2	HCV +	HCV +	+	Indeterminate
38–3540^e,n^	Indeterminate	–	–	+	ND	–	ND	ND	ND	+	+	ND	–	6	HCV +	HCV +	ND	Indeterminate
15–2479^e,o^	Indeterminate	ND	ND	–	–	ND	ND	ND	ND	+	ND	ND	ND	4	HCV +	HCV +	ND	Acetaminophen toxicity
10–2010^**a,p**^	Indeterminate	–	–	ND	–	ND	–	ND	ND	–	–	ND	+	38	HIV –	HIV –	–	Indeterminate
13–2273^**a,e,q**^	Indeterminate	–	–	ND	ND	ND	+	ND	ND	+	–	ND	+	4	HIV +	HIV +	ND	Indeterminate
13–3941^e,r^	Indeterminate	–	–	–	ND	ND	–	ND	ND	–	ND	–	–	7	CMV –, EBV +	CMV –, EBV +	ND	Multifactorial: CMV, EBV, lymphoma
37–4067	Indeterminate	–	–	ND	–	ND	+	ND	ND	–	ND	ND	ND	4	HPV type 159 +	HPV type 159 +	–	Indeterminate
13-2236^s^	Indeterminate	ND	ND	ND	ND	ND	ND	ND	ND	ND	ND	ND	ND	38	CMV +	CMV +	ND	CMV
20-2779^t^	Indeterminate	ND	–	–	–	ND	ND	ND	ND	–	–	ND	–	18	HHV-7 +	HHV-7 –	ND	Indeterminate
21–2824	Indeterminate	–	–	+	–	–	–	–	ND	–	ND	ND	–	16	HSV-1 +	HSV-1 +	ND	HSV-1
43–3569^u^	Indeterminate	–	–	ND	–	ND	ND	ND	ND	–	ND	ND	–	3	HSV-1 +	HSV-1 +	ND	HSV-1
43–3578^u^	Indeterminate	–	–	ND	ND	–	–	–	ND	–	–	ND	–	10	HSV-1 +	HSV-1 +	ND	HSV-1
18–2649	Indeterminate	–	–	–	–	ND	ND	ND	ND	–	ND	ND	ND	31	B19V +	B19V +	ND	B19V

Abbreviations: –, negative; +, positive; ALF, acute liver failure; anti-HBc, hepatitis B core antibody; anti-HBs, hepatitis B surface antibody; B19V, human parvovirus B19; CMV, cytomegalovirus; EBV, Epstein-Barr virus; HAV, hepatitis A virus; HBeAg, hepatitis B e antigen; HBsAg, hepatitis B surface antigen; HBV, hepatitis B virus; HCV, hepatitis C virus; HDV, hepatitis D virus; HEV, hepatitis E virus; HHV, human herpesvirus; HIV, human immunodeficiency virus; HPV, human papillomavirus; HSV, herpes simplex virus; IgM, immunoglobulin M; mNGS, metagenomic next-generation sequencing; NAT, nucleic acid testing; ND, not done; UCSF, University of California, San Francisco; Ultrio, Procleix Ultrio assay for simultaneous detection of HIV types 1/2 and HBV and HCV nucleic acids (Grifols Diagnostic Solutions and Hologic).

^a^Immunosuppressed patients, either HIV-positive (10–2010 and 13–2273) or currently being treated with steroids (15–2455) or steroids and mycophenolate mofetil (18–2653).

^b^After adjudication by Acute Liver Failure Study Group Causality Committee.

^c^Positive control samples.

^d^HSV-1 immunoglobulin M (IgM)/immunoglobulin G (IgG) positive, HSV-2 IgM positive, HSV-2 IgG negative, CMV IgG positive, EBV IgG positive.

^e^Tested positive for a viral infection by clinical site testing, although classified as indeterminate.

^f^HSV-1/HSV-2 IgG negative.

^g^Known to be chronically infected with HBV; unclear whether HBV infection was the cause of ALF and death, and thus was classified as indeterminate.

^h^APAP (*N*-acetyl-p-aminophenol/acetaminophen) adduct negative.

^i^APAP adduct negative.

^j^Negative for HBsAg and acetaminophen toxicity was thought to be a likely cause for patient’s ALF (although adducts not measured); thus, was classified as indeterminate.

^k^Positive for HBsAg, although HBV DNA was not done; clinical site may have misadjudicated this case as indeterminate.

^l^HSV-1/HSV-2 IgG negative.

^m^Known chronic HCV positive.

^n^Known chronic HCV positive; HSV-1/HSV-2 IgG positive; HSV-1/HSV-2 IgM negative; anti-EBV negative; anti-CMV negative.

^o^APAP-adducts positive.

^p^Possible lymphoma, on HIV medications, EBV PCR negative; HSV-1/HSV-2 PCR negative.

^q^APAP adduct negative.

^r^Known lymphoma; CMV DNA quantitative polymerase chain reaction (PCR) 2680 copies/mL, EBV quantitative PCR 194000 copies/mL, EBV anti-Epstein Barr nuclear antigen (EBNA) IgG positive, EBV anti-viral capsid antigen (VCA) IgM negative.

^s^Evidence of CMV and EBV exposure; heterophile agglutinin negative, CMV antibody positive, CMV antigen positive, EBV anti-EBNA IgG positive, VCA IgM negative, anti-HSV-1 positive.

^t^Re-extraction and repeat mNGS testing confirmed presence of HHV-7.

^u^Samples 43-3569 and 43-3578 were pooled together for mNGS analysis.

**Figure 2.**
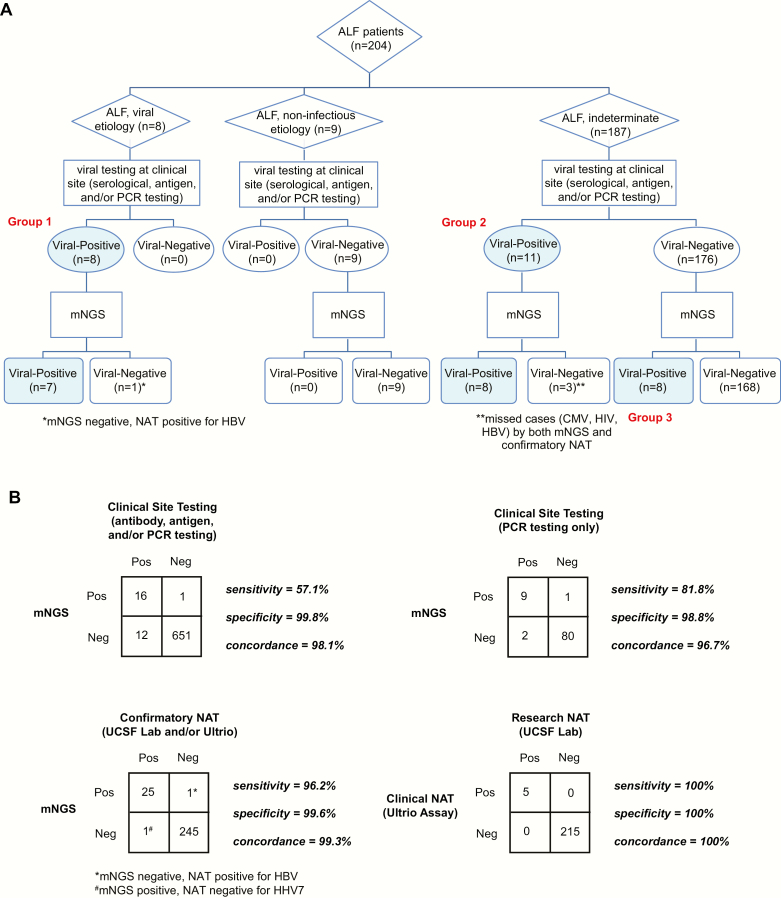
Testing of serum samples from 204 patients with acute liver failure (ALF) by metagenomic next-generation sequencing (mNGS). *A*, Flowchart of ALF cases subdivided into viral, noninfectious, and indeterminate etiologies. All cases positive for a pathogenic virus or discrepant between mNGS and clinical site testing were confirmed by virus-specific nucleic acid testing (NAT). *B*, Contingency tables in a 2 × 2 format comparing the relative performance of mNGS, clinical site testing, and confirmatory NAT (clinical Ultrio and research UCSF lab NAT) in the detection of viral pathogens. Abbreviations: ALF, acute liver failure; CMV, cytomegalovirus; HBV, hepatitis B virus; HIV, human immunodeficiency virus; mNGS, metagenomic next-generation sequencing; NAT, nucleic acid testing; Neg, negative; PCR, polymerase chain reaction; Pos, positive; UCSF, University of California, San Francisco; Ultrio, Procleix Ultrio assay for simultaneous detection of HIV types 1/2 and HBV and HCV nucleic acids (Grifols Diagnostic Solutions and Hologic).

Blinded mNGS analysis yielded 27 serum samples positive for a pathogenic virus by NGS, classified into 3 groups (Figure 2A; Table 2). First, 7 of 8 (87.5%) positive controls containing HAV or HBV were identified correctly. The single missed positive control had only 2 HBV sequences and was not called positive by mNGS at the predetermined thresholds, although it was subsequently positive for HBV by NAT. Another HBV-positive control was found to be coinfected by HSV-1. The second group comprised 11 cases that tested positive for viral infection by serology, PCR, or both, but were considered indeterminate by the site investigator due to residual uncertainty regarding the true etiology. Metagenomic sequencing confirmed 8 of these 11 cases (72.7%), failing to detect a case of HIV, a case of CMV in the context of CMV/EBV coinfection, and a case of HBV in mixed HBV, HCV, and HDV coinfection. However, in each of these 3 discrepant cases, the serum was also NAT negative for the missed virus, indicating that the viral nucleic acid may have been degraded or that the initial positive detection had been made using a method other than nucleic acid detection by mNGS or NAT (eg, serology). Finally, previously unrecognized viral infections were found in 8 cases: HSV-1 alone in 3 cases, and 1 case each of HBV, parvovirus B19, HHV-7, CMV, and HPV-159, a cutaneous betapapillomavirus [24]. The clinical relevance of betapapillomavirus detection by NGS is unknown, as these cutaneous HPV types are part of the normal skin flora [24], so likely represent contamination introduced during venipuncture. Importantly, 9 negative control samples with noninfectious etiologies for ALF were all virus negative by mNGS and clinical site testing. On a per-test, per-virus basis, mNGS results were 98.1% and 99.3% concordant with clinical site and confirmatory NAT testing, respectively (Figure 2B).

### Identification of Resistance Mutations

Consensus genomes for each of the viruses identified by mNGS were obtained by mapping the reads to the closest matched viral genome in the NCBI NT database and assembling a complete or partial consensus genome (Table 3). Ten HBV, HCV, and HSV-1 strains with complete sequence coverage of the relevant viral genes (eg, thymidine kinase and polymerase genes for HSV-1 [25]) were analyzed for resistance mutations. The analysis revealed an insertion in codon 145 of the thymidine kinase gene in 1 HSV-1 strain resulting in a frameshift mutation, suggesting possible resistance to acyclovir [25, 26], and an NS3(174S) polymorphism in 1 HCV strain, suggesting possible resistance to telaprevir [27, 28]. No previously described resistance mutations were detected in the remaining 8 strains.

**Table 3.** Metagenomic Assembly, Genotyping, and Mutation Analyses of Acute Liver Failure Virus-Positive Samples

Sample ID	Metagenomic Next-Generation Sequencing Result	No. of Reads	% Coverage	% Pairwise Identity	Resistance Mutations^b^
10–2006	Hepatitis A virus (HAV)	69368	99.6	97.7	–
13–2207	Hepatitis A virus (HAV)	11805	72.1	98.3	–
15–2455	Hepatitis A virus (HAV)	7778	95.8	97.2	–
23–2911	Hepatitis A virus (HAV)	57557	95.5	97.6	–
14–2440	Hepatitis B virus genotype A (HBV-A)	1395	58.2	99.1	Not detected
18–2653	Hepatitis B virus genotype B (HBV-B)	244005	100.0	97.2	Not detected
33–3251	Hepatitis B virus genotype D (HBV-D)	50420	100.0	95.2	Not detected
33–3251	Human herpesvirus 1 (herpes simplex virus type 1 [HSV-1])	104341	83.3	96.4	TK(145ins)c
10–2053	Hepatitis B virus genotype A (HBV-A)	2375	90.4	94.4	Not detected
21-2833	Hepatitis B virus genotype B (HBV-B)	100	34.9	96.3	
13–2383	Hepatitis B virus genotype C (HBV-C)	20947	97.4	96.7	Not detected
35–3354	Hepatitis B virus genotype D (HBV-D)	17901	100.0	96.6	Not detected
17–2605	Hepatitis B virus genotype A (HBV-A)	520	47.6	97.2	
11–2071	Hepatitis C virus subtype 1a (HCV-1a)	77	3.4	97.6	–
10–3875	Hepatitis C virus subtype 2b (HCV-2b)	8173	93.9	96.6	Not detected
38–3540	Hepatitis C virus subtype 3a (HCV-3a)	17413	91.0	97.8	Not detected
15–2479	Hepatitis C virus subtype 1a (HCV-1a)	14322	98.2	95.9	NS3 (174S)^d^
11–2071	Hepatitis D virus (HDV)	366900	93.2	94.6	–
13–2273	Human immunodeficiency virus 1 (HIV-1)	29	10.0	98.2	–
13–3941	Human herpesvirus 4 (Epstein-Barr virus)	162	4.3	94.9	–
37–4067	Human papillomavirus type 159	22	3.9	97.6	–
13–2236	Human herpesvirus 5 (cytomegalovirus)	837	15.3	96.8	–
20–2779	Human herpesvirus 7 (HHV-7)	6	0.2	97.2	–
21–2824	Human herpesvirus 1 (HSV-1)	1688	37.7	88.3	–
43–3569^a^	Human herpesvirus 1 (HSV-1)	27346	–	–	–
43–3578^a^	Human herpesvirus 1 (HSV-1)	27346	–	–	–
18–2649	Human parvovirus B19	41969	94.9	96.2	–

Abbreviation: –, not reported.

^a^Samples 43-3569 and 43-3578 were pooled and sequenced together, so the read count, percentage coverage, percentage pairwise identity, and resistance mutations for each individual sample cannot be determined. The total HSV-1 read count for the pool is reported.

^**b**^Resistance information is reported when there is >90% coverage of the target viral gene.

^c^Possible resistance to acyclovir.

^d^Possible resistance to telaprevir.

## DISCUSSION

In the current study, we used a metagenomic sequencing approach to identify potential viral pathogens in 204 adult patients with ALF collected by the United States ALFSG. A total of 187 patients (92%) were clinically defined as indeterminate, while the remaining patients represented blinded controls for either hepatitis A or B infection, or established noninfectious causes of ALF. The overall concordance of mNGS relative to clinical site and confirmatory NAT testing was high at 98.1% and 99.3%, respectively (Figure 2B). Previously unrecognized viruses of likely or potential clinical significance, including HBV, HSV-1, parvovirus B19, CMV, and HHV-7, were identified in serum samples from 8 patients (4.3%). Importantly, no sequences corresponding to novel viruses were detected.

Notably, the mNGS analyses identified several cases of dual or triple infections with multiple viruses. These included 1 case of HBV/HSV-1 coinfection, 1 case of CMV/EBV coinfection, and 1 triple infection with HBV, HCV, and HDV. The patient with HBV/HSV-1 coinfection had a markedly elevated alanine aminotransferase (ALT) level of 7280 units per liter (U/L) [normal range 8–48 U/L] and died within 21 days of study admission; coinfection with 2 hepatotropic viruses may have resulted in a rapidly progressive, more fulminant disease course. The case of coinfection with CMV and EBV occurred in the setting of a patient with known lymphoma, and reactivation by these 2 herpesviruses may have precipitated ALF. Interestingly, the case of triple coinfection was associated with hepatitis B surface antigen antigenemia but undetectable HBV viral loads (PCR and mNGS negativity). However, triple coinfection with HBV, HCV, and HDV has been reported to be associated with increased liver damage and severe chronic disease [29].

HSV-1 infection was identified in 4 of 187 (2.1%) indeterminate ALF patients by mNGS, subsequently confirmed by PCR. All HSV-1 cases had not been previously recognized by clinical site testing, as HSV testing is not routinely ordered in the initial workup of ALF. These results contrast with those from a prior smaller study, also from the ALFSG cohort, that found no new cases of HSV in 51 indeterminate patients by PCR [5]. The 4 additional cases of HSV-1 detected in the current study may have been unmasked by screening of a greater number of patients, as differences in rates of detection were not significant (*P* = .58 by 2-tailed Fisher exact test).

Although HSV-1 has been described as an extremely rare cause of ALF, the prevalence of mNGS- and PCR-positive cases in patients with indeterminate ALF in the current study (2.1%) and generally poor clinical outcomes [30] suggest that HSV-1 should be considered as part of the early workup, even if the potential of inadvertent contamination or reactivation (unrelated to ALF) from HSV-1 cannot be ruled out. The 4 immunocompetent patients who were positive for systemic HSV by mNGS consisted of 2 men and 2 women between 18 years and 62 years of age, none of whom had a history of liver disease. The 2 HSV-1 infected patients with extremely high aspartate aminotransferase (AST)/ALT enzyme levels (7000–16000 U/L) died during the initial hospitalization. The 2 remaining patients had milder elevations (400–2000 U/L) and either survived or were of unknown clinical status at the 21-day follow-up. Three of the 4 patients had normal white blood cell (WBC) counts, except for 1 patient who was coinfected with HBV and had elevated WBC counts of 29.4 × 10^9^ cells/L (normal, 3.5–10 × 10^9^ cells/L).

We also found a case positive for parvovirus B19 infection among 187 cases of indeterminate ALF, versus none in a previous study [4]. Acute parvovirus B19 infections are largely asymptomatic or cause flu-like symptoms [31], but in rare instances can cause acute hepatitis [32], including a reported case of parvovirus-associated hepatic failure requiring liver transplantation [33]. Our patient with parvovirus B19 infection was a 75-year-old man on no outpatient medications who presented with ALF of unknown etiology (AST, 1194 U/L [normal range 7–55 U/L]; ALT, 1172 U/L [normal range 8–48 U/L]; alkaline phosphatase, 124 U/L [normal range 45–115 U/L]; bilirubin, 20.5 mg/dL [normal range 0.1–1.2 mg/dL]; international normalized ratio, 2.0 [normal range 0.8–1.1]; and albumin, 2 g/dL [normal range 3.5–5.0 g/dL]). The patient tested negative for HAV, HBV, and HCV serology, and mNGS did not reveal any viral infections apart from parvovirus B19. Bacterial and fungal blood cultures were negative, as was a toxicology screen. The patient eventually developed multiorgan failure and died 21 days postadmission. In the absence of an alternative diagnosis, we believe that parvovirus B19 was the likely cause of ALF in this patient.

HHV-7 was also detected by mNGS in a single case. This finding is of unclear clinical significance, as acute HHV-7 infection is often asymptomatic and >90% of individuals are seropositive by adulthood [34]. Nevertheless, acute hepatitis from primary HHV-7 infection has been previously described in an infant, with viral DNA detected in the liver and subsequent seroconversion [35]. For the 2 identified cases of HIV-1, neither patient was coinfected with HBV or HCV, which can accelerate cirrhosis and liver decompensation, or, for HBV, precipitate ALF by viral reactivation in the setting of medication withdrawal [36, 37]. Thus, the etiology of ALF in the 2 HIV-1–infected patients remains to be established, although 1 patient may have developed ALF secondary to HIV-associated lymphoma.

The sparse detection rate of infections from a pathogenic virus (7 of 187 [3.2%]) in indeterminate ALF cases by mNGS is unlikely due to decreased sensitivity, as the sensitivity between mNGS and “gold standard” confirmatory NAT was 96.2% (Figure 2B). Rather, the low yield of viruses in the current study suggests that either most cases of ALF are due to noninfectious causes, such as acetaminophen toxicity [38], or that analysis of invasively acquired samples, such as liver biopsy (not available in the current study), may be needed to boost diagnostic sensitivity.

Clinical mNGS testing is likely to become part of a routine diagnostic workup for acute infectious diseases such as hepatitis [9, 19, 39, 40]. Unlike multiplex PCR, which targets a predefined panel of microorganisms, mNGS can interrogate clinical samples for any and all pathogens simultaneously. Thus, limited material is not expended by following the traditional diagnostic paradigm of serial testing for a priori targeted infectious agents, which can be expensive, time-consuming, and low-yield. However, rigorous assessment of the performance characteristics of mNGS testing in the clinical laboratory can be challenging, although validation efforts are now under way at multiple sites, including ours [41]. Given its high specificity, an mNGS clinical assay may be useful in the near future not only for direct diagnosis but also as a test to exclude (“rule out”) infection.

In conclusion, the indeterminate ALF patient group represents a heterogeneous mix, comprised of patients for whom diagnostic testing may be incomplete leading to unclear or “no apparent” diagnosis, as well as those with dual/triple diagnoses or ambiguous results. Testing for rare and unexpected viral infections is not performed in routine clinical practice, as specific antiviral treatments are currently limited. Nevertheless, HSV testing does appear to be of value, as 2.1% (4 of 187) of indeterminate ALF cases were found to be positive, disease progression may be unusually rapid with HSV infection, and prompt treatment with antiviral medications such acyclovir can be life-saving. It is also reassuring that the use of mNGS for comprehensive screening of 187 indeterminate ALF patients yielded only 7 additional cases of infection from viral pathogens and no novel viruses. These results suggest that standard clinical testing for the hepatitis A–E viruses, supplemented by additional targeting of uncommon viruses such as HSV and parvovirus B19, is sufficient to screen for blood-borne viral infections associated with ALF, at least until more comprehensive diagnostic technologies such as mNGS become clinically available.

## Supplementary Data

Supplementary materials are available at *Clinical Infectious Diseases* online. Consisting of data provided by the authors to benefit the reader, the posted materials are not copyedited and are the sole responsibility of the authors, so questions or comments should be addressed to the corresponding author.

## Supplementary Material

Supplementary_Methods

Supplementary_Tables

## Notes

***Financial support.*** This work was supported by the National Institutes of Health (NIH) (grant number R01 HL105704 to C. Y. C.); the National Institute of Diabetes and Digestive and Kidney Diseases (NIDDK) (grant number U01-DK-58369 to W. M. L. for ALFSG); and an Abbott Viral Discovery Award (to C. Y. C.). The NIDDK Central Repositories supplied the samples from the United States Acute Liver Failure Study reported here.

***Potential conflicts of interest.*** C. Y. C. is the director of the UCSF-Abbott Viral Diagnostics and Discovery Center and receives research funding from Abbott Laboratories. W. M. L. receives research support from Merck and Conatus for pharmaceutical studies unrelated to acute liver failure. C. Y. C. and S. N. N. are inventors of patent PCT/US156/52912 titled “Pathogen Detection Using Next-Generation Sequencing” pending with regards to the SURPI+ computational pipeline. All other authors report no potential conflicts. All authors have submitted the ICMJE Form for Disclosure of Potential Conflicts of Interest. Conflicts that the editors consider relevant to the content of the manuscript have been disclosed.
