# Supplementary material for: Viral Surveillance in Serum Samples From Patients With Acute Liver Failure By Metagenomic Next-Generation Sequencing
Source: Clin Infect Dis. 2017 Jul 19;65(9):1477–85. doi: 10.1093/cid/cix596 (PMC5848299; doi:10.1093/cid/cix596)
Supplement: Supplementary_Methods [file cix596_suppl_supplementary-methods.docx]

**SUPPLEMENTARY METHODS**

**The Acute Liver Failure Study Group (ALFSG) cohort**

Patients were selected from a nationwide cohort of 1,848 adult ALF consecutive patients who were enrolled in a large data and biosample registry from 23 tertiary care centers within the US between January 1998 and October 2010. Inclusion criteria defining ALF were the presence of coagulopathy (PT > 15 seconds or INR >1.5 IU) and any grade of hepatic encephalopathy (HE) that had occurred within 26 weeks of initial onset of symptoms. Patients with chronic liver disease (CLD) or cirrhosis were excluded, with the exception of certain patients with chronic hepatitis B or Wilson disease presenting with rapid onset of severe illness in the absence of a history of known liver disease. For each enrolled subject, detailed demographic, clinical, laboratory, radiologic, and outcomes data were recorded. Etiological diagnoses were made by the principal investigator at each study site using standard criteria (and routinely reviewed by ALFSG clinical leadership), based on the history and clinical presentation, laboratory, radiographic information and liver biopsy results, when available. When a thorough investigation failed to disclose a cause, the etiology was designated “Indeterminate”. In some instances, serological data to complete the exclusion of other etiologies were not available. For each subject, written informed consent was obtained from next of kin because of the presence of hepatic encephalopathy, with approval consent revisited with the patient after recovery. All centers were in compliance with their local institutional review board requirements.

**Genome assembly, genotyping, and identification of resistance mutations**

Directed assembly of partial or complete genomes in ALF samples positive for a pathogenic virus was performed in Geneious [1], using the genome sequence of the closest matched reference sequence identified by SURPI+ as a scaffold. . For >70% complete consensus genomes, phenotypic drug resistance was predicted using the Geno2pheno program for HBV and HCV (<http://hcv.bioinf.mpi-inf.mpg.de/index.php>) [2, 3], or manual sequence comparison to a database of resistance-related mutations in the thymidine kinase and DNA polymerase genes for HSV [4]. Raw metagenomic sequence data with removal of human / primate reads identified by SURPI+ (by SNAP and Bowtie2 alignment to the primate-annotated sequences in NCBI NT) have been deposited in the NCBI Sequence Read Archive (SRA) (accession number SRP109147).

**REFERENCES**

1. Kearse M, Moir R, Wilson A, et al. Geneious Basic: an integrated and extendable desktop software platform for the organization and analysis of sequence data. Bioinformatics **2012**; 28(12): 1647-9.

2. Beerenwinkel N, Daumer M, Oette M, et al. Geno2pheno: Estimating phenotypic drug resistance from HIV-1 genotypes. Nucleic Acids Res **2003**; 31(13): 3850-5.

3. Welsch C, Domingues FS, Susser S, et al. Molecular basis of telaprevir resistance due to V36 and T54 mutations in the NS3-4A protease of the hepatitis C virus. Genome Biol **2008**; 9(1): R16.

4. Sauerbrei A, Bohn-Wippert K, Kaspar M, Krumbholz A, Karrasch M, Zell R. Database on natural polymorphisms and resistance-related non-synonymous mutations in thymidine kinase and DNA polymerase genes of herpes simplex virus types 1 and 2. J Antimicrob Chemother **2016**; 71(1): 6-16.
